# Prenatal maternal infections and early childhood developmental outcomes: analysis of linked administrative health data for Greater Glasgow & Clyde, Scotland

**DOI:** 10.1111/jcpp.14028

**Published:** 2024-06-27

**Authors:** Iain Hardie, Aja Murray, Josiah King, Hildigunnur Anna Hall, Emily Luedecke, Louise Marryat, Lucy Thompson, Helen Minnis, Philip Wilson, Bonnie Auyeung

**Affiliations:** ^1^ Department of Psychology, School of Philosophy, Psychology and Language Sciences University of Edinburgh Edinburgh UK; ^2^ Centre for Health Security and Communicable Disease Control Directorate of Health Reykjavík Iceland; ^3^ School of Health Sciences University of Dundee Dundee UK; ^4^ Centre for Rural Health, Institute of Applied Health Sciences University of Aberdeen Aberdeen UK; ^5^ Gillberg Neuropsychiatry Centre University of Gothenburg Gothenburg Sweden; ^6^ School of Health and Wellbeing University of Glasgow Glasgow UK; ^7^ Centre for Research and Education in General Practice University of Copenhagen Copenhagen Denmark

**Keywords:** Child development, maternal factors, prenatal, infection, CNS

## Abstract

**Background:**

Previous research has linked prenatal maternal infections to later childhood developmental outcomes and socioemotional difficulties. However, existing studies have relied on retrospectively self‐reported survey data, or data on hospital‐recorded infections only, resulting in gaps in data collection.

**Methods:**

This study used a large linked administrative health dataset, bringing together data from birth records, hospital records, prescriptions and routine child health reviews for 55,856 children born in Greater Glasgow & Clyde, Scotland, 2011–2015, and their mothers. Logistic regression models examined associations between prenatal infections, measured as both hospital‐diagnosed prenatal infections and receipt of infection‐related prescription(s) during pregnancy, and childhood developmental concern(s) identified by health visitors during 6‐8 week or 27‐30 month health reviews. Secondary analyses examined whether results varied by (a) specific developmental outcome types (gross‐motor‐skills, hearing‐communication, vision‐social‐awareness, personal‐social, emotional‐behavioural‐attention and speech‐language‐communication) and (b) the trimester(s) in which infections occurred.

**Results:**

After confounder/covariate adjustment, hospital‐diagnosed infections were associated with increased odds of having at least one developmental concern (OR: 1.30; 95% CI: 1.19–1.42). This was broadly consistent across all developmental outcome types and appeared to be specifically linked to infections occurring in pregnancy trimesters 2 (OR: 1.34; 95% CI: 1.07–1.67) and 3 (OR: 1.33; 95% CI: 1.21–1.47), that is the trimesters in which foetal brain myelination occurs. Infection‐related prescriptions were not associated with any clear increase in odds of having at least one developmental concern after confounder/covariate adjustment (OR: 1.03; 95% CI: 0.98–1.08), but were associated with slightly increased odds of concerns specifically related to personal‐social (OR: 1.12; 95% CI: 1.03–1.22) and emotional‐behavioural‐attention (OR: 1.15; 95% CI: 1.08–1.22) development.

**Conclusions:**

Prenatal infections, particularly those which are hospital‐diagnosed (and likely more severe), are associated with early childhood developmental outcomes. Prevention of prenatal infections, and monitoring of support needs of affected children, may improve childhood development, but causality remains to be established.

## Introduction

Maternal health during pregnancy plays an important role in later childhood development. One important aspect of this is maternal infections, which occur commonly during pregnancy and can require treatment with prescription drugs, or hospital admission in more severe cases (Collier et al., 2009; WHO Global Maternal Sepsis Study Research Group, 2020). Animal models of prenatal maternal infection suggest that a mother's immune response to an infectious agent (i.e. cytokine signalling, antibody production), which is known as maternal immune activation (MIA), creates a cascade of events that can impact upon foetal brain development (Garay, Hsiao, Patterson, & McAllister, 2013; Massrali, Adhya, Srivastava, Baron‐Cohen, & Kotter, 2022; Oskvig, Elkahloun, Johnson, Phillips, & Herkenham, 2012). Whilst more research is needed, human studies tend to be consistent with this (see Han, Patel, Jones, & Dale, 2021).

Most studies examining prenatal infections and childhood development have focussed on links between prenatal infections and formal diagnoses of neurodevelopmental conditions in children. For example, some research suggests prenatal infections are associated with diagnosis of autism in children, particularly among severe maternal infections involving hospitalisation as these involve higher levels of MIA (Atladottir et al., 2010; Jiang et al., 2016; Lee et al., 2015). Meanwhile, other research has examined potential links between prenatal infections and attention‐deficit/hyperactivity disorder (ADHD), with mixed results (e.g. see Ginsberg et al., 2019; Mann & McDermott, 2011; Werenberg Dreier et al., 2016). Prenatal infections have also been linked to mental health conditions in adults such as schizophrenia and bipolar disorder (Cordeiro, Tsimis, & Burd, 2015; Khandaker, Zimbron, Lewis, & Jones, 2013; Parboosing, Bao, Shen, Schaefer, & Brown, 2013).

Whilst most studies focus on neurodevelopmental or mental health conditions, other research into the relationship between prenatal infections and a broader range of early childhood developmental outcomes (i.e. not necessarily involving any formal diagnoses) is also important. This is because many conditions are not diagnosed until late childhood or adulthood, and understanding outcomes early can help ensure that any potential support needs are met as quickly and effectively as possible. To date, only a few pieces of research on this have been conducted. One study of linked administrative data from New South Wales, Australia, found hospital‐diagnosed prenatal maternal infections to be associated with increased odds of social, emotional, physical, cognitive and communication developmental vulnerabilities in children at age 5 (Green et al., 2018). Similarly, a cohort study using data from Boston and Providence, USA, found prenatal bacterial infections to be associated with reduced cognitive performance at age 7 (Lee, Papandonatos, Savitz, Heindel, & Buka, 2020). However, other research, conducted using data from a UK cohort study, found no association between hospital‐recorded prenatal infections and childhood socioemotional developmental outcomes at age 3, but did find self‐reported maternal infections to be associated with increased emotional problems (Hall, Speyer, Murray, & Auyeung, 2021). Finally, research by Kwok, Hall, Murray, Lombardo, and Auyeung (2022), which examined maternal infections in each trimester of pregnancy and associations with child cognitive outcomes, suggests that infections in the third trimester could have an effect on cognitive abilities later in childhood (although effect sizes were small).

There are, however, some important limitations to these existing pieces of research. Notably, they have tended to use data on prenatal infections that are either (a) self‐reported, and thus limited by issues relating to a lack of a strong verification that infections were present and by the retrospective nature of reporting (see Hall et al., 2021; Kwok et al., 2022), or (b) based on hospital records only, and thus limited by only including a minority of infections that are most severe (Green et al., 2018). The present study overcomes some of these limitations by making use of prenatal infections data from both hospital records and infection‐related prescriptions. These were linked to data on birth records and routine child health reviews to form a large linked administrative health dataset covering children born in 2011–2015 in Greater Glasgow & Clyde, Scotland. This dataset was used to address the following aims:

**Aim 1:** To examine associations between prenatal maternal infections, measured as both hospital‐diagnosed prenatal infections and receipt of infection‐related prescription(s) during pregnancy, and having childhood developmental concern(s) identified by health visitors during early routine child health reviews.
**Aim 2:** To examine whether these associations vary by (a) specific developmental outcome types (i.e. gross‐motor‐skills, hearing‐communication, vision‐social‐awareness, personal‐social, emotional‐behavioural‐attention, and speech‐language‐communication development) or (b) the trimester(s) in which infections occurred.

## Methods

### Data and participants

**Figure 1:**
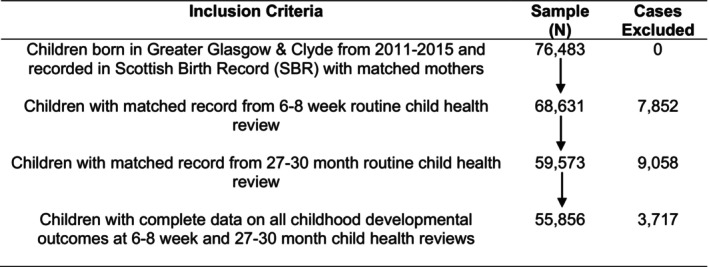
Flowchart of sample selection procedure

The linked administrative health dataset – made up of data from hospital records, birth records, prescriptions and child health reviews – included children born in the National Health Service (NHS) health board region of Greater Glasgow & Clyde, 2011–2015. This included all children who had linked maternal health records and complete records of child developmental outcomes from child health reviews conducted (routinely and universally) at ages 6 to 8 weeks and 27–30 months (for full details of child health reviews, see Public Health Scotland, 2020; Scottish Government, 2015). In total, this gave a final sample of 55,856 children and their mothers. A flowchart providing full details of the sample selection procedure, and exclusions, is provided in Figure 1.

The exclusions detailed in Figure 1 relate to the fact that although child health reviews are universal in that they are targeted at all children, they are not taken up by, or recorded for, everyone. In particular, the following groups are all excluded for not having outcome data: (a) those whose parents/carers chose not to take up the reviews, (b) those who health visitors were unable to make contact with, (c) those who were born in NHS Greater Glasgow & Clyde but who moved outside of Scotland before becoming old enough for the reviews, or (d) those who took part in the reviews but did not have developmental outcomes assessed.

### Childhood developmental outcome measures

The first outcome variable was having any (i.e. at least one) childhood developmental concerns identified by health visitors during the 6‐8 week or 27‐30 month child health review of each child in the sample (coded: 0 = ‘No’, 1 = ‘Yes’). These could relate to either gross‐motor‐skills, hearing‐communication or vision‐social‐awareness development measured at 6‐8 week reviews, or personal‐social, emotional‐behavioural‐attention, or speech‐language‐communication development measured at 27‐30 month reviews. Developmental concerns were defined as cases where at least one of the above developmental observations were categorised by health visitors as ‘abnormal’, ‘a concern’ or ‘doubtful’ (i.e. suggestive of a possible abnormality, see: Playford, Dibben, & Williamson, 2017) rather than as ‘normal’ or ‘no concerns’. Health visitors make these observations based on any/all of the following: (a) elicitation of parental concerns, (b) a structured observation of the child and/or (c) the results of an ‘Ages and Stages’ questionnaire or other validated developmental assessment questionnaires (see Scottish Government, 2015).

In addition, a further set of outcome variables were used in which the previous overall developmental concerns variable was disaggregated in order to indicate whether each child in the sample had developmental concerns identified that related specifically to (a) gross‐motor‐skills, (b) hearing‐communication, (c) vision‐social‐awareness, (d) personal‐social, (e) emotional‐behavioural‐attention and (f) speech‐language‐communication development. These outcomes were examined as six binary variables (each coded: 0 = ‘No’, 1 = ‘Yes’).

### Prenatal maternal infection measures

Two primary measures of prenatal maternal infection were used as explanatory variables in the analysis. The first was hospital‐diagnosed prenatal infection(s), which indicates whether, for each mother in the sample, Scotland's ‘general/acute inpatient and day case’ hospital records (see Information Services Division Scotland, 2022d) or ‘maternity inpatient and day case’ hospital records (see Information Services Division Scotland, 2022a) show the diagnosis of any maternal infection during the month of childbirth or in one of the nine preceding months (coded: 0 = ‘No’, 1 = ‘Yes’). Infections were identified using ICD10 codes. As the mechanism(s) behind the relationship between prenatal infections and child development outcomes is still not fully understood (e.g. whether the mechanism is related to infection or associated inflammation), a deliberately broad definition of hospital‐diagnosed prenatal infection was used, with all ICD10 codes that were assessed as being related to either an infection or an inflammation being included. All postpartum/puerperal infections were excluded as although these were listed in maternity hospital records they would have occurred after delivery so are not prenatal infections. A full list of the ICD10 codes that were included is provided in Table S1.

The second primary measure used was receipt of infection‐related prescription(s), which indicates whether, for each mother in the sample, Scotland's Prescribing Information System (PIS, see Information Services Division Scotland, 2022b) records show any receipt of prescriptions that are likely to have been for an infection during the month of childbirth or in one of the preceding nine months (coded: 0 = ‘No’, 1 = ‘Yes’). This measure was used in order to indicate potential infection rather than to look at whether the drugs themselves may have potential effects on child development (this is based on the assumption that GPs or other physicians would not prescribe drugs known to be unsafe during pregnancy except in the rare event that the expected benefits were considered greater than any potential risks to the foetus). Like in existing literature making similar use of PIS data related to infections (e.g. Scott et al., 2018), infection‐related prescriptions were defined as prescriptions included in British National Formulary (BNF) Chapter 5: Infections (British National Formulary, 2023). A full list of drugs/prescriptions included is provided in Table S2.

In addition to the above primary explanatory variables, two further sets of explanatory variables were used in order to examine whether any potential associations between prenatal infections and childhood developmental outcomes varied depending on the trimester in which infections occurred. This is important to consider because foetal brain myelination does not begin until the second trimester of pregnancy (with further growth reinforcements occuring in trimester 3, see Cordeiro et al., 2015; Kwok et al., 2022). To measure this, the hospital‐diagnosed prenatal infection(s) measure was disaggregated into (a) hospital‐diagnosed prenatal infection(s) in trimester 1, (b) hospital‐diagnosed prenatal infection(s) in trimester 2 and (c) hospital‐diagnosed prenatal infection(s) in trimester 3. Similarly, the receipt of infection‐related prescription measure was disaggregated into (a) receipt of infection‐related prescription(s) during pregnancy trimester 1, (b) receipt of infection‐related prescription(s) during pregnancy trimester 2 and (c) receipt of infection‐related prescription(s) during pregnancy trimester 3.

Finally, additional sensitivity analysis also included alternative measures of hospital‐diagnosed prenatal infections and receipt of infection‐related prescriptions, whereby infections/prescriptions from during the month of childbirth were excluded. These measures were included because infections from during the month of childbirth are often those picked up when mothers are in hospital for the actual delivery/birth, Importantly, including this type of infection meant the inclusion of infections like Group B Strep carrier cases and herpes simplex virus encephalitis picked up when mothers are in hospital for delivery. These may be harmful for babies but are unlikely to lead to an immune response in mothers that would affect foetal development.

### Confounders and covariates

The analysis also included a number of potential confounders and covariates as control variables. Maternal age and area‐based deprivation were included as confounders, as these factors are known to be potentially associated with both maternal health during pregnancy and childhood health and developmental outcomes (Glick, Kadish, & Rottenstreich, 2021; Lean, Derricott, Jones, & Heazell, 2017; Reijneveld, Brugman, Verhulst, & Verloove‐Vanhorick, 2005; Vinikoor‐Imler, Messer, Evenson, & Laraia, 2011; Yasumitsu‐Lovell et al., 2023). Information on maternal age came from maternity hospital admissions data and it was defined as maternal age at time of birth (coded: 0 = ≤19, 1 = 20–35, 2 = ≥36, which is in line with the ‘optimal’ (20–35) and ‘suboptimal’ (≤19/≥36) maternal age categorisations for neurodevelopment identified by previous research (Yasumitsu‐Lovell et al., 2023)). Information on area‐based deprivation came from data on Scottish Index of Multiple Deprivation (SIMD), which was calculated from home postcodes and categorised into quintiles (coded: 1 = most deprived, 2 = more deprived, 3 = medium deprived, 4 = less deprived and 5 = least deprived).

In addition, sex of child, maternal history of mental health hospital admissions and maternal prenatal smoking were included as covariates as they are potentially heavily associated with childhood development (Green et al., 2018; National Advisory Council on Women and Girls, 2022; Wehby, Prater, McCarthy, Castilla, & Murray, 2011). Sex of child was defined as a binary variable (coded: 0 = male, 1 = female), with data on this coming from Scottish birth records. Data on maternal history of mental health hospital admissions came from hospital records on mental health inpatient and day case hospital admissions (see Information Services Division Scotland, 2022c). It was also defined as a binary variable (coded 0 = no history of admissions, 1 = history of admissions, i.e. one or more recorded admissions). Data were not available on less severe maternal mental health issues (i.e. those not requiring admission to hospital). Finally, maternal prenatal smoking was defined as a binary indicator of whether mothers smoked during pregnancy (coded: 0 = no, 1 = yes), and this information came from maternity hospital admissions data.

### Statistical analysis

The analysis used logistic regression models to measure associations between the prenatal maternal infection measures (including the primary measures, secondary measures and sensitivity analysis measures outlined above) and childhood developmental outcome measures (i.e. having any developmental concerns identified and concerns identified specifically relating to gross‐motor‐skills, hearing‐communication, vision‐social‐awareness, personal‐social, emotional‐behavioural‐attention and speech‐language‐communication development).

Modelling was conducted using a hierarchical approach, which involved (a) unadjusted models, (b) models which adjust for confounders only (i.e. maternal age and area‐based deprivation) and (c) models which additionally adjust for covariates (i.e. sex of child, maternal history of mental health admissions and maternal prenatal smoking in addition to maternal age and area‐based deprivation). The analysis was carried out as a complete case analysis because, unlike with standardised developmental outcomes like SDQ scores (see SDQ Info, 2024), health visitor judgements on child developmental outcomes do not have set rules for imputing missing values, and due to the size of the study dataset, it would be too computationally demanding to impute missing values.

All analyses were conducted using Stata/MP 16 software within Scotland's National Safe Haven, with access to all datasets being facilitated by the electronic Data Research and Innovation Service (eDRIS) team at Public Health Scotland. The analysis plan for this study was preregistered using Open Science Framework (available here: https://osf.io/dx4vb), and the reporting in this study is consistent with ‘REporting of studies Conducted using Observational Routinely collected health Data’ (RECORD) guidelines (see Benchimol et al., 2015).

## Results

### Descriptive statistics

Descriptive statistics showing the characteristics of the sample, in terms of prevalence of study variables, are provided in Table 1. Overall, 21.2% of children in the sample had at least one developmental concern identified by health visitors. This is broadly in line with previous estimates of early developmental concerns in Greater Glasgow & Clyde using similar datasets, although slightly higher than levels reported at a Scottish population level (Information Services Division, 2017; Scottish Government, 2023). Of these, concerns were more commonly identified for outcomes measured at 27‐ to 30‐month health reviews, particularly those relating to speech‐language‐communication development (for which 13.1% of the sample had concerns identified) and emotional‐behavioural‐attention development (for which 10.5% of sample had concerns identified). Conversely, developmental concerns at 6‐ to 8‐week child health reviews (i.e. gross‐motor‐skills, hearing‐communication and vision‐social‐awareness development) were rare, occurring for just 0.5%–1.8% of sample.

**Table 1:** Descriptive statistics for childhood developmental outcomes, prenatal infections and confounders/covariates

	*N*	%
Primary childhood developmental outcome		
Any (i.e. at least one) childhood developmental concerns identified by health visitors		
No	44,026	78.8
Yes	11,830	21.2
Secondary childhood developmental outcomes		
Developmental concern: gross‐motor‐skills development (6–8 weeks)		
No	54,852	98.2
Yes	1,004	1.8
Developmental concern: hearing‐communication development (6–8 weeks)		
No	55,598	99.5
Yes	258	0.5
Developmental concern: vision‐social‐awareness development (6–8 weeks)		
No	55,210	98.8
Yes	646	1.2
Developmental concern: personal‐social development (27–30 months)		
No	53,160	95.2
Yes	2,696	4.8
Developmental concern: emotional‐behavioural‐attention development (27–30 months)		
No	50,003	89.5
Yes	5,853	10.5
Developmental concern: speech‐language‐communication development (27–30 months)		
No	48,487	86.8
Yes	7,369	13.1
Primary prenatal infection measures		
Hospital‐diagnosed prenatal infection		
No	52,999	94.9
Yes	2,857	5.1
Receipt of infection‐related prescription(s) during pregnancy		
No	40,774	73.0
Yes	15,082	27.0
Secondary prenatal infection measures		
Hospital‐diagnosed prenatal infection in trimester 1		
No	55,539	99.4
Yes	317	0.5
Hospital‐diagnosed prenatal infection in trimester 2		
No	55,449	99.3
Yes	407	0.7
Hospital‐diagnosed prenatal infection in trimester 3		
No	53,600	96.0
Yes	2,256	4.0
Receipt of infection‐related prescription(s) during pregnancy trimester 1		
No	50,047	89.6
Yes	5,809	10.4
Receipt of infection‐related prescription(s) during pregnancy trimester 2		
No	49,850	89.3
Yes	6,006	10.7
Receipt of infection‐related prescription(s) during pregnancy trimester 3		
No	46,980	84.1
Yes	8,876	15.9
Sensitivity analysis prenatal infection measures		
Hospital‐diagnosed prenatal infection (excluding month of childbirth)		
No	54,369	97.3
Yes	1,487	2.7
Receipt of infection‐related prescription(s) during pregnancy (excluding month of childbirth)		
No	43,059	77.1
Yes	12,797	22.9
Confounders/Covariates		
Maternal age at time of birth		
<20	2,472	4.4
20–35	44,524	79.7
>35	8,860	15.9
SIMD quintile		
1 (most deprived)	22,245	39.8
2 (more deprived)	10,329	18.5
3 (medium deprived)	8,452	15.1
4 (less deprived)	7,161	12.8
5 (least deprived)	7,669	13.7
Sex of child		
Male	28,348	50.7
Female	27,508	49.3
Maternal history of mental health hospital admissions		
No	54,970	98.4
Yes	886	1.6
Maternal prenatal smoking		
No	47,908	85.8
Yes	7,948	14.2

5.1% of mothers in the sample had records of hospital‐diagnosed infections, and 27.0% had records of receiving infection‐related prescriptions during pregnancy, although these figures were lower (2.7% and 22.9%, respectively) when infections/prescriptions from the month of childbirth were excluded (i.e. in the sensitivity analysis prenatal infection measures). With regard to confounders/covariates, it is noteworthy that maternal history of mental health admissions was fairly rare (1.6% of sample) and that the sample tended to be from more deprived SIMD quintiles, which reflects the fact that Glasgow has relatively high levels of deprivation (see Understanding Glasgow, 2020). In addition to Table 1, further descriptive statistics showing (a) variation in prenatal infection measures and confounders/covariates across childhood developmental outcomes, and (b) variation in childhood developmental outcomes and confounders/covariates across prenatal infection measures, are provided in Tables S3 and S4.

### Prenatal infections and having at least one childhood developmental concern

Results showing associations between prenatal infections and having any (i.e. at least one) childhood developmental concerns identified by health visitors at 6–8 weeks or 27–30 months are provided in Table 2 (fully adjusted results for key variables only) and Table S5a,b (unadjusted, confounder adjusted and fully adjusted results for all variables). The results suggest that hospital‐diagnosed prenatal infections were associated with increased odds of developmental concerns (OR: 1.43; 95% CI: 1.31–1.55 in unadjusted model and OR: 1.33; 95% CI: 1.22–1.45 in confounder adjusted model). Even after adjustment for confounders and covariates, they were associated with a 1.30 (95% CI: 1.19–1.42) increase in the odds of having at least one childhood developmental concern. Meanwhile, receipt of infection‐related prescription(s) during pregnancy was associated with a small increase in the odds of having at least one developmental concern in the unadjusted model (OR: 1.10; 95% CI: 1.05–1.16), but this was largely attenuated in the confounder adjusted (OR: 1.03; 95% CI: 0.98–1.08) and fully adjusted (OR: 1.03; 95% CI: 0.98–1.08) models.

**Table 2:** Odds ratios (95% CIs) for fully adjusted associations between prenatal infection(s) and having any (i.e. at least one) childhood developmental concern identified by health visitors

	Having any (i.e. at least one) childhood developmental concern identified
Hospital‐diagnosed prenatal infection(s)	
[No]	
Yes	1.30*** (1.19–1.42)
Receipt of infection‐related prescription(s) during pregnancy	
[No]	
Yes	1.03 (0.98–1.08)

Models fully adjust for maternal age at time of birth, SIMD quintile, sex of child, maternal history of mental health hospital admissions and maternal prenatal smoking. Reference categories are shown in square brackets. Childhood developmental concerns include those measured at both 6‐ to 8‐week and 27‐ to 30‐month routine child health visits.

*p < .05, **p < .01 and ***p < .001.

### Prenatal infections and specific types of childhood developmental concerns

Results showing associations between prenatal infections and having specific types of childhood developmental concerns are shown in Table 3 (fully adjusted results for key variables only) and Table S6a,b (unadjusted, confounder adjusted and fully adjusted results for all variables). The results suggest that, even after adjustment for confounders and covariates, hospital‐diagnosed prenatal infections were associated with increased odds of childhood developmental concerns relating to gross‐motor‐skills (OR:1.30; 95% CI: 1.01–1.67), hearing‐communication (OR: 1.33; 95% CI: 0.82–2.17) and vision‐social‐awareness (OR: 1.46; 95% CI: 1.08–1.96) development at age 6–8 weeks, and personal‐social (OR: 1.34; 95% CI: 1.15–1.56), emotional‐behavioural‐attention (OR: 1.36; 95% CI: 1.22–1.52) and speech‐language‐communication (OR: 1.33; 95% CI: 1.20–1.47) development at age 27–30 months.

**Table 3:** Odds ratios (95% CIs) for fully adjusted associations between prenatal infections and having specific types of childhood developmental concerns identified by health visitors

	Type of childhood developmental concern identified					
	6‐ to 8‐week child health review			27‐ to 30‐month child health review		
	Gross‐motor skills	Hearing‐communication	Vision‐social awareness	Personal‐social	Emotional‐behavioural‐attention	Speech‐language‐communication
Hospital‐diagnosed prenatal infection(s)						
[No]						
Yes	1.30* (1.01–1.67)	1.33 (0.82–2.16)	1.46* (1.08–1.96)	1.34*** (1.15–1.56)	1.36*** (1.22–1.52)	1.33*** (1.20–1.47)
Receipt of infection‐related prescription(s) during pregnancy						
[No]						
Yes	1.05 (0.91–1.21)	1.08 (0.82–1.43)	0.92 (0.77–1.10)	1.12* (1.03–1.22)	1.15*** (1.08–1.22)	0.97 (0.92–1.03)

Models fully adjust for maternal age at time of birth, SIMD quintile, sex of child, maternal history of mental health hospital admissions and maternal prenatal smoking. Reference categories are shown in square brackets.

*p < .05, **p < .01 and ***p < .001.

Meanwhile, with regard to infection‐related prescriptions the results were less clear. Infection‐related prescriptions did appear to be associated with a small increase in the odds of personal‐social (OR: 1.12; 95% CI: 1.03–1.22 in fully adjusted model) and emotional‐behavioural‐attention development (OR: 1.15; 95% CI: 1.08–1.23 in fully adjusted model). However, there were no clear associations for any of the other types of developmental concerns examined.

### Prenatal infections, by trimester and having at least one childhood developmental concern

Results showing associations between prenatal infections, by trimester and having at least one childhood developmental concern are provided in Table 4 (fully adjusted results for key variables only) and Table S7a,b (unadjusted, confounder adjusted and fully adjusted results for all variables). They highlight that the positive association between hospital‐diagnosed prenatal infections and having at least one childhood developmental concern appeared to be specifically linked to infections occurring in trimesters 2 (OR: 1.34; 95% CI: 1.07–1.67 in fully adjusted model) and 3 (OR: 1.33; 95% CI: 1.21–1.47 in fully adjusted model). There was not a clear association between hospital‐diagnosed prenatal infections in trimester 1 and having at least one childhood developmental concern (OR: 1.11; 95% CI: 0.84–1.45 in fully adjusted model). In terms of infection‐related prescriptions, there did not appear to be much variation in the relationship between their receipt and having childhood developmental concern(s) by trimester.

**Table 4:** Odds ratios (95% CIs) for fully adjusted associations between prenatal infections, by trimester and having any (i.e. at least one) childhood developmental concern identified by health visitors

	Having any (i.e. at least one) childhood developmental concern identified
Trimester 1 hospital‐diagnosed prenatal infection(s)	
[No]	
Yes	1.11 (0.84–1.45)
Trimester 2 hospital‐diagnosed prenatal infection(s)	
[No]	
Yes	1.34* (1.07–1.67)
Trimester 3 hospital‐diagnosed prenatal infection(s)	
[No]	
Yes	1.33*** (1.21–1.47)
Trimester 1 receipt of infection‐related prescription(s) during pregnancy	
[No]	
Yes	1.09* (1.02–1.16)
Trimester 2 receipt of infection‐related prescription(s) during pregnancy	
[No]	
Yes	1.06* (1.00–1.14)
Trimester 3 receipt of infection‐related prescription(s) during pregnancy	
[No]	
Yes	1.04 (0.99–1.10)

Models fully adjust for maternal age at time of birth, SIMD quintile, sex of child, maternal history of mental health hospital admissions and maternal prenatal smoking. Reference categories are shown in square brackets. Childhood developmental concerns include those measured at both 6‐ to 8‐week and 27‐ to 30‐month routine child health visits.

*p < .05, **p < .01 and ***p < .001.

### Sensitivity analysis

The results of the sensitivity analysis, which repeated the main analysis but excluding hospital‐diagnosed infections and infection‐related prescriptions from the month of childbirth, are provided in Table S8a,b. The results were broadly in line with the results of the main analysis.

## Discussion

This study used linked administrative health data to examine relationships between prenatal infections, measured as both hospital‐diagnosed infections and receipt of infection‐related prescriptions, and early childhood developmental outcomes among children born in NHS Greater Glasgow & Clyde, Scotland, 2011–2015. The findings suggest that, after adjusting for measured confounders and covariates, hospital‐diagnosed prenatal infections were associated with increased odds of children having at least one developmental concern identified during 6‐8 week or 27‐30‐month routine child health reviews. This relationship was broadly consistent across all developmental outcome types measured and appeared to be specifically linked to infections occurring in trimesters 2 and 3 of pregnancy. The findings also suggest that receipt of infection‐related prescriptions during pregnancy was associated with increased odds of children having at least one childhood developmental concern, but this was attenuated after accounting for confounders and covariates. There were, however, increased odds of developmental concerns specifically related to personal‐social and emotional‐behavioural‐attention development even after confounder and covariate adjustment. The relationship between receipt of infection‐related prescriptions and developmental outcomes did not appear to vary by the trimester in which prescriptions were received.

Overall, these findings suggest that MIA, or some other mechanism arising from significant prenatal infections, is associated with increased likelihood of adverse childhood developmental outcomes. This is in line with what is observed in animal models of maternal prenatal infections (Garay et al., 2013; Massrali et al., 2022; Oskvig et al., 2012). In addition, the research findings of the present study suggest that prenatal infections diagnosed in hospital had a particularly clear association with developmental outcomes. This is in line with the results of some previous human studies such as Green et al. (2018), but not others, for example Hall et al. (2021). This most likely reflects the fact that infections picked up in hospital will typically be more severe, and more severe infections will, in general, involve higher levels of MIA (see Hall, Willis, Rodriguez, & Schwarz, 2023; Jiang et al., 2016). In addition, the fact that this association appeared to be specifically related to infections in the second and third trimester of pregnancy most likely reflects the fact that foetal brain myelination occurs in the second and third trimester, and therefore, this is the period in which MIA may have the greatest effect on prenatal brain development (see Cordeiro et al., 2015; Kwok et al., 2022).

One novel aspect of this study is that, whilst previous studies on prenatal infections and childhood development have relied on retrospectively self‐reported survey data, or data on hospital‐recorded infections only, this study was able to also use data on receipt of infection‐related prescriptions as an additional indicator of prenatal infection. The finding that receipt of infection‐related prescriptions during pregnancy was associated with increased odds of developmental concerns related to personal‐social and emotional‐behavioural‐attention development backs up previous research that found maternal self‐reported prenatal infections to be associated with childhood emotional problems (Hall et al., 2021). However, the infection‐related prescription measure used in the present study overcomes some of the limitations that this previous research had, in particular, limitations related to its self‐reported data potentially lacking strong verification that an infection was present and problems arising from the retrospective nature of its reporting (see Hall et al., 2021, p. 1647).

The key strength of this study was that it was able to make use of a large linked administrative health dataset, which included data on multiple indicators of prenatal infections, and a range of childhood developmental outcomes. Nevertheless, there are some limitations that must be noted. First, it was beyond the scope of this study to examine variations in outcomes based on whether infections were bacterial, viral or other infection types. Moreover, whilst a range of confounders and covariates were controlled for, it is possible that there are additional confounding factors that were not observed in the dataset. For example, possible unobserved confounding mechanisms might be disruption of antenatal attachment caused by infection, some genetic confounding linking susceptibility to infection, or to a general propensity to receive antibiotic prescriptions, and neurodevelopmental outcomes. In particular, given the subjective nature of antibiotic prescribing (see Borek et al., 2020; Public Health England, 2015), it is possible that unmeasured confounders with a genetic component (e.g. mild anxiety) might explain at least part of the observed association between infection‐related prescriptions (specifically antibiotic prescriptions) during pregnancy and poorer child personal‐social and emotional‐behavioural‐attention developmental outcomes (see Howie & Bigg, 1980). Also, given that childhood developmental outcomes are heritable, it is possible that parental behaviours related to their own neurodevelopmental outcomes could impact a mother's likelihood of receiving a diagnosis of infection, for example by affecting their propensity to consult their GP about a possible infection.

In addition, there are also some limitations related to the administrative health dataset used in this study's analysis. It is well documented that studies using administrative health data are often not fully representative of the target population due to missing data arising from people failing to interact with health services (e.g. due to barriers such as housing problems, living in remote areas or a wide range of other reasons) or due to inaccuracies in data linkage processes (see Harron et al., 2017; Shaw et al., 2022). In the case of the present study specifically, it was conducted as a complete case analysis, and participation in Scotland's universal health visiting pathway (i.e. the routine child health reviews in which child developmental outcomes were measured) is known to be lower among those living in more deprived areas (Horne, Marryat, & Wood, 2021). This means that the study participants may not be completely representative of all children born in Greater Glasgow & Clyde during the analysis period. More broadly, Greater Glasgow & Clyde is also unlikely to be representative of Scotland (or the United Kingdom), as it is a mostly urban area and has relatively high levels of deprivation (Understanding Glasgow, 2020).

The analysis outlined in this study used hospital‐diagnosed prenatal infections and receipt of infection‐related prescriptions as the main prenatal infection indicators. As discussed above, this provides a fuller picture of infections than previous studies. However, each of these indicators comes with their own set of strengths and weaknesses. The strength of using hospital‐diagnosed infections is that they provide a strong verification of the presence of an infection, as patients will have been thoroughly examined before a diagnosis is made. However, infections diagnosed in hospital likely include only a small proportion of all infections, typically made up of more severe cases only. Moreover, there can be nuances in how hospital‐diagnosed infections are coded and in how medical notes/records are interpreted by the medical reviewers entering ICD10 codes (Hashimoto, Brodt, Skelly, & Dettori, 2014). On the other hand, infection‐related prescriptions cover a much larger number of prenatal infections, as PIS data cover all medicines that are prescribed and dispensed in the community (Information Services Division Scotland, 2022b). However, its accuracy in capturing infections may be affected by geographical or temporal variations in GP prescribing practices (see Devine, O'Kane, & Bucholc, 2021; Guthrie et al., 2022; Pouwels, Dolk, Smith, Smieszek, & Robotham, 2018b), and there have been known issues relating to inappropriate prescribing and overprescribing of antibiotics in the United Kingdom and elsewhere (e.g. see Pouwels, Dolk, Smith, Robotham, & Smieszek, 2018a; Smieszek et al., 2018).

To conclude, although this study has some limitations, by including data on both hospital‐diagnosed infections and infection‐related prescriptions it provides as full a picture as possible on prenatal infections. The study provides evidence that maternal infections during pregnancy, particularly those which are (a) hospital‐diagnosed (i.e. likely more severe) and (b) occurring in the second and third trimester of pregnancy, are associated with early childhood developmental outcomes in children. As this may be linked to the process of MIA affecting foetal brain development, prevention of prenatal infections in the second and third trimester of pregnancy, alongside monitoring of support needs of affected children, may improve childhood development. However, the findings require confirmation in experimental study designs, and an important area for future research is analyses separate by infection pathogen (i.e. bacterial infection, viral infection and other infection types) as there may be difference between pathogens.

## Ethical considerations

This project was approved by the research ethics committee for the School of Philosophy, Psychology and Language Sciences (PPLS) at the University of Edinburgh (reference: 190‐1718/2). The project also received information governance approval from the NHS Scotland Public Benefit and Privacy Panel for Health and Social Care (HSC‐PBPP, reference: 1617–0314). Written consent from participants was not required or obtained. All data used in this study were housed within Scotland's National Safe Haven, coordinated by the electronic Data Research and Innovation Service (eDRIS) team at Public Health Scotland. The data were only accessed by approved researchers.

**Key points**

- Previous studies suggest that prenatal infections, and the maternal immune activation that comes with them, are associated with child developmental outcomes. However, research to date has been based on infection data that is either self‐reported or included infections diagnosed in hospital only.
- This study examined associations between prenatal infections, measured by both hospital‐diagnosed infections and receipt of infection‐related prescriptions, and child developmental concerns identified by health visitors at ages 6–8 weeks and 27–30 months.
- Hospital‐diagnosed prenatal infections were consistently associated with developmental concerns. Maternal receipt of infection‐related prescriptions during pregnancy was also associated with developmental concerns, but only those related to personal‐social and emotional‐behavioural‐attention development.
- This suggests that prenatal infections, particularly severe infections, are associated with early childhood developmental outcomes.

## Supporting information


**Table S1.** List of ICD10 codes included in this study's hospital‐diagnosed prenatal infections definition.
**Table S2.** List of drugs/prescriptions included in this study's receipt of infection‐related.
**Table S3.** Descriptive statistics for prenatal infections and confounders/covariates by childhood developmental outcome(s).
**Table S4.** Descriptive statistics for childhood developmental outcomes and confounders/covariates by prenatal infections.
**Table S5a.** Odds ratios (95% CIs) for unadjusted, confounder adjusted and fully adjusted associations between hospital‐diagnosed prenatal infections and having any (i.e. at least one) childhood developmental concerns identified by health visitors.
**Table S5b.** Odds ratios (95% CIs) for unadjusted, confounder adjusted and fully adjusted associations between receipt of infection‐related prescription(s) during pregnancy and having any (i.e. at least one) childhood developmental concerns identified by health visitors.
**Table S6a.** Odds ratios (95% CIs) for unadjusted, confounder adjusted and fully adjusted associations between hospital‐diagnosed prenatal infections and having specific types of childhood developmental concerns identified by health visitors.
**Table S6b.** Odds ratios (95% CIs) for unadjusted, confounder adjusted and fully adjusted associations between receipt of infection‐related prescription(s) during pregnancy and having specific types of childhood developmental concerns identified by health visitors.
**Table S7a.** Odds ratios (95% CIs) for unadjusted, confounder adjusted and fully adjusted associations between hospital‐diagnosed prenatal infections, by trimester, and having any (i.e. at least one) childhood developmental concerns identified by health visitors.
**Table S7b.** Odds ratios (95% CIs) for unadjusted, confounder adjusted and fully adjusted associations between receipt of infection‐related prescription(s), by trimester, and having any (i.e. at least one) childhood developmental concerns identified by health visitors.
**Table S8a.** Odds ratios (95% CIs) for fully adjusted associations between prenatal infections (with month of childbirth excluded) and having any (i.e. at least one) adverse childhood development outcome.
**Table S8b.** Odds ratios (95% CIs) for fully adjusted associations between prenatal infections (with month of childbirth excluded) and having a specific type of adverse childhood development outcome.

## Data Availability

The administrative health datasets used for this study are not publicly available, but can be accessed via successfully applying to the NHS Scotland Public Benefit and Privacy Panel for Health and Social Care (HSC‐PBPP). The authors of the present study were supported in applying for approval from HSC‐PBPP by the electronic Data Research and Innovation Service (eDRIS) team at Public Health Scotland. eDRIS also facilitated access to the data via Scotland's National Safe Haven.
