# Supplementary material for: Prenatal maternal infections and early childhood developmental outcomes: analysis of linked administrative health data for Greater Glasgow & Clyde, Scotland
Source: J Child Psychol Psychiatry. 2024 Jun 27;66(1):30–40. doi: 10.1111/jcpp.14028 (PMC11652418; doi:10.1111/jcpp.14028)
Supplement: Supplementary file 1 — Table S1. List of ICD10 codes included in this study's hospital‐diagnosed prenatal infections definition. Table S2. List of drugs/prescriptions included in this study's receipt of infection‐related. Table S3. Descriptive statistics for prenatal infections and confounders/covariates by childhood developmental outcome(s). Table S4. Descriptive statistics for childhood developmental outcomes and confounders/covariates by prenatal infections. Table S5a. Odds ratios (95% CIs) for unadjusted, confounder adjusted and fully adjusted associations between hospital‐diagnosed prenatal infections and having any (i.e. at least one) childhood developmental concerns identified by health visitors. Table S5b. Odds ratios (95% CIs) for unadjusted, confounder adjusted and fully adjusted associations between receipt of infection‐related prescription(s) during pregnancy and having any (i.e. at least one) childhood developmental concerns identified by health visitors. Table S6a. Odds ratios (95% CIs) for unadjusted, confounder adjusted and fully adjusted associations between hospital‐diagnosed prenatal infections and having specific types of childhood developmental concerns identified by health visitors. Table S6b. Odds ratios (95% CIs) for unadjusted, confounder adjusted and fully adjusted associations between receipt of infection‐related prescription(s) during pregnancy and having specific types of childhood developmental concerns identified by health visitors. Table S7a. Odds ratios (95% CIs) for unadjusted, confounder adjusted and fully adjusted associations between hospital‐diagnosed prenatal infections, by trimester, and having any (i.e. at least one) childhood developmental concerns identified by health visitors. Table S7b. Odds ratios (95% CIs) for unadjusted, confounder adjusted and fully adjusted associations between receipt of infection‐related prescription(s), by trimester, and having any (i.e. at least one) childhood developmental concerns identified by health visitors. Table [file JCPP-66-30-s001.doc]

# **Supporting Information**

**Table S1.** List of ICD10 codes included in this study’s hospital-diagnosed prenatal infections definition.

| **ICD10 Codes** | **Type of Infections Included** |
| --- | --- |
| A/B | virus infections; bacterial infections; sexually transmitted infections/diseases |
| G0/G531/G630/ G940 | meningitis; cranial nerve infections; polyneuropathy infections; hydrocephalus infections |
| H0/H1/H20/H22/H320/H440/H441/H451/H481 | eye infections |
| I30/I310/I311/I32/ I33/I38/I39/I40/  I41/I430/I520/I521/I681/I980/I981 | pericarditis; endocarditis; myocarditis; heart infections; artery infections; cardiovascular infections |
| J0/J1/J2?J31/J32/J35/J36/J37/  J39/J40/J41/J42/J440/J85/J86 | sinusitis; tonsillitis; laryngitis; common cold; influenza; pneumonia; respiratory infections; bronchitis; rhinitis; pharyngitis; peritonsillar abscess; pharynx infections; pulmonary disease with infection; gangrene/lung abscess; pyothorax |
| K04/K05/K102/K112/K113/K122/K140/K35/K61/K630/K65/K67/K75/K770/K81/K930 | periodontitis; pulpitis; gingivitis; necrosis; jaw inflammation/infection; salivary gland infection; cellulitis/mouth abscess; glossitis; appendicitis; anal infection/abscess; intestine abscess/infection; peritonitis; liver infection; cholecystitis; tuberculous of intestine |
| L0 | skin infections |
| M0 | sepctic arthitis |
| N080/N10/N11/N12/N22/N290/N291/N30/N330/N34/N390/N61/N7 | glomerular disorders involving infection; interstitial nephritis; urinary calculus infection; kidney infection; cystitis; urethritis; urinary tract infection unspecified; infection in breast; pelvic/vaginal/vulva infection or inflammation |
| O23/O98 | infections/infectious disease associated with pregnancy |
| R50 | fever |
| T880 | Infection following immunisation |

**Table S2.** List of drugs/prescriptions included in this study’s receipt of infection-related.

| **Category of Drugs/**  **Prescriptions** | **Specific Drugs/Prescriptions Included** |
| --- | --- |
| Antibacterials (antibiotics) | -Penicillin (Amoxicillin, Ampicillin, Co-amoxiclav, Co-fluampicil, Flucloxacillin, Phenoxymethylpenicillin)  -Cephalosporins and other beta-lactams (Cefaclor, Cephalexin, Cefixime, Cefradine, Cefuroxime)  -Tetracyclines (Doxycycline, Lymecycline, Minocycline, Oxytetracycline, Clobetasone with Oxytetracycline and Nystatin, Tretracycline)  -Aminoglycosides (Gentamicin, Neomycin Sulfate, Betamethasone with Neomycin, Hydrocortisone with Neomycin, Triamcinolone Gramicidin Neoymycin and Nystatin, Dexamethasone with Neomycin and Polymyxin B)  -Macrolides (Azithromycin, Clarithromycin, Erythromycin, Erythromycin with Zinc Acetate, Isotretinoin with Erythromycin)  -Clindamycin and Lincomycin (clindamycin)  -Other Antibacterials (Chloramphenicol, Colistin, Fusidic Acid, Betamethason with Fusidic Acid, Hydrocortisone with Fusidic Acid, Vancomycin)  -Antituberculosis Drugs (Cycloserine, Ethambutol Hydrochloride, Rifampicin with Isoniazid, Rifampicin)  -Antileprotic Drugs (Dapsone)  -Metronidazole, Tinidazole and Ornidazole (Metronidazole, Tinidazole)  -Quinolones (Cifrofloxacin, Levofloxacin, Moxifloxacin, Norfloxacin, Ofloxacin)  -Urinary Tract Infection Drugs (Methenamine, Nitrofurantoin) |
| Antifungals | -Triazole Antifungals (Fluconazole, Fluconozole and Clotrimazole, Itraconazole)  -Imidazole Antifungals (Ketoconazole)  -Polyene Antifungals (Amphotericin, Nystatin)  -Other Antifungals (Griseofulvin) |
| Antivirals | -Herpesvirus Infections (Aciclovir, Famciclovir, Penciclovir, Valaciclovir)  -Viral Hepatitis (Enteclavir)  -Influenza (Amantadine Hydrochloride, Oseltamivir, Zanamivir) |
| Antiprotozoals | Antimalarial drugs (Mefloquine, Quinine, Proguanil Hydrochloride with Chloroquine Phosphate, Atovaquone with Proguanil Hydrochloride) |
| Antiprotozoals | -Drugs for Threadworms (Mebendazole, Piperazine with Senna) |

**Table S3. Descriptive statistics for prenatal infections and confounders/covariates by childhood developmental outcome(s).**

|  | **N (%)** | |
| --- | --- | --- |
|  | **Any (at least one) childhood developmental concerns** | |
|  | **No** | **Yes** |
| **Prenatal Infections** |  |  |
| Hospital-diagnosed prenatal infection(s) |  |  |
| *No* | 41,948 (79.2%) | 11,051 (20.8%) |
| *Yes* | 2,078 (72.7%) | 779 (27.3%) |
| Hospital-diagnosed prenatal infection(s) (trimester 1) |  |  |
| *No* | 43,788 (78.8%) | 11,751 (21.2%) |
| *Yes* | 238 (75.1%) | 79 (24.9%) |
| Hospital-diagnosed prenatal infection(s) (trimester 2) |  |  |
| *No* | 43,737 (78.9%) | 11,712 (21.1%) |
| *Yes* | 289 (71.0%) | 118 (29.0%) |
| Hospital-diagnosed prenatal infection(s) (trimester3) |  |  |
| *No* | 42,394 (79.1%) | 11,206 (20.9%) |
| *Yes* | 1,632 (72.3%) | 624 (27.7%) |
| Receipt of infection-related prescription(s) during pregnancy |  |  |
| *No* | 32,323 (79.3%) | 8,451 (20.7%) |
| *Yes* | 11,703 (77.6%) | 3,379 (22.4%) |
| Receipt of infection-related prescription(s) during pregnancy (trimester 1) |  |  |
| *No* | 39,606 (79.1%) | 10,441 (20.9%) |
| *Yes* | 4,420 (76.1%) | 1,389 (23.9%) |
| Receipt of infection-related prescription(s) during pregnancy (trimester 2) |  |  |
| *No* | 39,421 (79.1%) | 10,429 (20.9%) |
| *Yes* | 4,605 (76.7%) | 1,401 (23.3%) |
| Receipt of infection-related prescription(s) during pregnancy (trimester 3) |  |  |
| *No* | 37,159 (79.1%) | 9,821 (20.9%) |
| *Yes* | 6,867 (77.8%) | 2,009 (22.6%) |
| **Confounders/Covariates** |  |  |
| Maternal age at time of birth |  |  |
| *<20* | 1,680 (68.0%) | 792 (32.0%) |
| *20-35* | 35,095 (78.8%) | 9,429 (21.2%) |
| *>35* | 7,251 (81.8%) | 1,609 (18.2%) |
| SIMD quintile |  |  |
| *1 (most deprived)* | 16,444 (73.9%) | 5,801 (26.1%) |
| *2 (more deprived)* | 8,041 (77.9%) | 2,288 (22.1%) |
| *3 (medium deprived)* | 6,844 (81.0%) | 1,608 (19.0%) |
| *4 (less deprived)* | 6,018 (84.0%) | 1,143 (16.0%) |
| *5 (least deprived* | 6,679 (87.1%) | 990 (12.9%) |
| Sex of child |  |  |
| *Male* | 20,644 (72.8%) | 7,704 (27.2%) |
| *Female* | 23,382 (85.0%) | 4,126 (15.0%) |
| Maternal history of mental health hospital admissions |  |  |
| *No* | 43,425 (79.0%) | 11,545 (21.0%) |
| *Yes* | 601 (67.8%) | 285 (32.2%) |
| Maternal prenatal smoking |  |  |
| *No* | 38,607 (80.6%) | 9,301 (19.4%) |
| *Yes* | 5,419 (68.2%) | 2,529 (31.8%) |

**Table S4. Descriptive statistics for childhood developmental outcomes and confounders/covariates by prenatal infections.**

|  | **N (%)** | | | |
| --- | --- | --- | --- | --- |
|  | **Hospital-recorded prenatal infection(s)** | | **Receipt of infection-related prescription(s) during pregnancy** | |
|  | **No** | **Yes** | **No** | **Yes** |
| **Primary Childhood developmental outcome** |  |  |  |  |
| Any (i.e. at least one) childhood developmental concerns identified by health visitors |  |  |  |  |
| *No* | 41,948 (95.4%) | 2,078 (4.7%) | 32,323 (73.4%) | 11,703 (26.6%) |
| *Yes* | 11,051 (93.4%) | 779 (6.6%) | 8,451 (71.4%) | 3,379 (28.6%) |
| **Secondary Childhood developmental outcomes** |  |  |  |  |
| Developmental concern: gross motor development (6-8 weeks) |  |  |  |  |
| *No* | 52,063 (94.9%) | 2,789 (5.1%) | 40,057 (73.0%) | 14,795 (27.0%) |
| *Yes* | 936 (93.2%) | 68 (6.8%) | 717 (71.4%) | 287 (28.6%) |
| Developmental concern: hearing-communication development (6-8 weeks) |  |  |  |  |
| *No* | 52,759 (94.9%) | 2,839 (5.1%) | 40,592 (73.0%) | 15,006 (27.0%) |
| *Yes* | 240 (93.0%) | 18 (6.9%) | 182 (70.5%) | 76 (29.5%) |
| Developmental concern: vision-social-awareness development (6-8 weeks) |  |  |  |  |
| *No* | 52,401 (94.9%) | 2,809 (5.1%) | 40,293 (73.0%) | 14,917 (27.0%) |
| *Yes* | 598 (92.6%) | 48 (7.4%) | 481 (74.5%) | 165 (25.6%) |
| Developmental concern: personal-social development (27-30 months) |  |  |  |  |
| *No* | 50,500 (95.0%) | 2,660 (5.0%) | 38,896 (73.2%) | 14,264 (26.8%) |
| *Yes* | 2,499 (92.7%) | 197 (7.3%) | 1,878 (69.7%) | 818 (30.3%) |
| Developmental concern: emotional-behavioural-attention development (27-30 months) |  |  |  |  |
| *No* | 47,573 (95.1%) | 2,430 (4.9%) | 36,748 (73.5%) | 13,255 (26.5%) |
| *Yes* | 5,426 (92.7%) | 427 (7.3%) | 4,026 (68.8%) | 1,827 (31.2%) |
| Developmental concern: speech-language-communication development (27-30 months) |  |  |  |  |
| *No* | 46,132 (95.1%) | 2,355 (4.9%) | 35,422 (73.1%) | 13,065 (26.9%) |
| *Yes* | 6,867 (93.2%) | 502 (6.8%) | 5,352 (72.6%) | 2,017 (27.4%) |
| **Confounders/Covariates** |  |  |  |  |
| Maternal age at time of birth |  |  |  |  |
| *<20* | 2,252 (91.1%) | 220 (8.9%) | 1,179 (47.7%) | 1,293 (52.3%) |
| *20-35* | 42,283 (95.0%) | 2,241 (5.0%) | 32,626 (73.3%) | 11,898 (26.7%) |
| *>35* | 8,464 (95.5%) | 396 (4.5%) | 6,969 (78.7%) | 1,891 (21.3%) |
| SIMD quintile |  |  |  |  |
| *1 (most deprived)* | 20,906 (94.0%) | 1,339 (6.0%) | 15,694 (70.6%) | 6,551 (29.4%) |
| *2 (more deprived)* | 9,778 (94.7%) | 551 (5.3%) | 7,423 (71.9%) | 2,906 (28.1%) |
| *3 (medium deprived)* | 8,052 (95.3%) | 400 (4.7%) | 6,197 (73.3%) | 2,255 (26.7%) |
| *4 (less deprived)* | 6,820 (95.2%) | 341 (4.8%) | 5,427 (75.8%) | 1,734 (24.2%) |
| *5 (least deprived* | 7,443 (97.1%) | 226 (2.9%) | 6,033 (78.7%) | 1,636 (21.3%) |
| Sex of child |  |  |  |  |
| *Male* | 26,878 (94.8%) | 1,470 (5.2%) | 20,753 (73.2%) | 7,595 (26.8%) |
| *Female* | 26,121 (95.0%) | 1,387 (5.0%) | 20,021 (72.8%) | 7,487 (27.2%) |
| Maternal history of mental health hospital admissions |  |  |  |  |
| *No* | 52,191 (94.9%) | 2,779 (5.1%) | 40,172 (73.1%) | 14,798 (26.9%) |
| *Yes* | 808 (91.2%) | 78 (8.8%) | 602 (67.9%) | 284 (32.1%) |
| Maternal prenatal smoking |  |  |  |  |
| *No* | 45,629 (95.2%) | 2,279 (4.8%) | 35,219 (73.5%) | 12,689 (26.5%) |
| *Yes* | 7,370 (92.7%) | 578 (7.3%) | 5,555 (73.0%) | 2,393 (30.1%) |

**Table S5a.** Odds ratios (95% CIs) for unadjusted, confounder adjusted and fully adjusted associations between hospital-diagnosed prenatal infections and having any (i.e. at least one) childhood developmental concerns identified by health visitors.

|  | **Having any (i.e. at least one) childhood developmental concerns identified** | | |
| --- | --- | --- | --- |
|  | Unadjusted | Confounder adjusted | Fully adjusted |
| **Hospital-diagnosed prenatal infection(s)** |  |  |  |
| *[No]* |  |  |  |
| *Yes* | 1.43*** (1.31-1.55) | 1.33*** (1.22-1.45) | 1.30*** (1.19-1.42) |
| **Maternal age at time of birth** |  |  |  |
| *<20* |  | 1.55*** (1.42-1.69) | 1.49*** (1.35-1.63) |
| *[20-35]* |  |  |  |
| *>35* |  | 0.94* (0.88-1.00) | 0.94 (0.89-1.00) |
| **SIMD quintile** |  |  |  |
| *1 (most deprived)* |  | 1.46*** (1.28-1.56) | 1.39*** (1.30-1.48) |
| *2 (more deprived)* |  | 1.20*** (1.11-1.29) | 1.18*** (1.10-1.27) |
| *[3 (medium deprived)]* |  |  |  |
| *4 (less deprived)* |  | 0.81*** (0.75-0.89) | 0.84*** (0.77-0.91) |
| *5 (least deprived)* |  | 0.64*** (0.59-0.70) | 0.67*** (0.61-0.73) |
| **Sex of child** |  |  |  |
| *[Male]* |  |  |  |
| *Female* |  |  | 0.46*** (0.44-0.48) |
| **Maternal history of mental health hospital admissions** |  |  |  |
| *[No]* |  |  |  |
| *Yes* |  |  | 1.48*** (1.28-1.72) |
| **Maternal prenatal smoking** |  |  |  |
| *[No]* |  |  |  |
| *Yes* |  |  | 1.64*** (1.55-1.73) |

*Notes*: Reference categories are shown in square brackets. Childhood developmental outcomes include those measured at both 6-8 weeks and 27-30 months routine child health visits. *p<0.05, **p<0.01, ***p<0.001.

**Table S5b.** Odds ratios (95% CIs) for unadjusted, confounder adjusted and fully adjusted associations between receipt of infection-related prescription(s) during pregnancy and having any (i.e. at least one) childhood developmental concerns identified by health visitors.

|  | **Having any (i.e. at least one) childhood developmental concerns identified** | | |
| --- | --- | --- | --- |
|  | Unadjusted | Confounder adjusted | Fully adjusted |
| **Receipt of infection-related prescription(s) during pregnancy** |  |  |  |
| *[No]* |  |  |  |
| *Yes* | 1.10*** (1.05-1.16) | 1.03 (0.98-1.08) | 1.03 (0.98-1.08) |
| **Maternal age at time of birth** |  |  |  |
| *<20* |  | 1.56*** (1.42-1.70) | 1.49*** (1.36-1.63) |
| *[20-35]* |  |  |  |
| *>35* |  | 0.94* (0.89-1.00 | 0.94 (0.89-1.00) |
| **SIMD quintile** |  |  |  |
| *1 (most deprived)* |  | 1.47*** (1.38-1.56) | 1.39*** (1.31-1.48) |
| *2 (more deprived)* |  | 1.20*** (1.12-1.29) | 1.18*** (1.10-1.27) |
| *[3 (medium deprived)]* |  |  |  |
| *4 (less deprived)* |  | 0.82*** (0.75-0.88) | 0.84*** (0.77-0.91) |
| *5 (least deprived)* |  | 0.64*** (0.59-0.70) | 0.67*** (0.61-0.73) |
| **Sex of child** |  |  |  |
| *[Male]* |  |  |  |
| *Female* |  |  | 0.46*** (0.44-0.48) |
| **Maternal history of mental health hospital admissions** |  |  |  |
| *[No]* |  |  |  |
| *Yes* |  |  | 1.49*** 1.29-1.73) |
| **Maternal prenatal smoking** |  |  |  |
| *[No]* |  |  |  |
| *Yes* |  |  | 1.64*** (1.55-1.74) |

*Notes*: Reference categories are shown in square brackets. Childhood developmental concerns include those measured at both 6-8 weeks and 27-30 months routine child health visits. *p<0.05, **p<0.01, ***p<0.001.

**Table S6a. Odds ratios (95% CIs) for unadjusted, confounder adjusted and fully adjusted associations between hospital-diagnosed prenatal infections and having specific types of childhood developmental concerns identified by health visitors.**

|  | **Type of childhood developmental concerns identified** | | | | | | | | | | | | | | | | | |
| --- | --- | --- | --- | --- | --- | --- | --- | --- | --- | --- | --- | --- | --- | --- | --- | --- | --- | --- |
|  | **6-8 weeks child health review** | | | | | | | | | **27-30 months child health review** | | | | | | | | |
|  | **Gross motor** | | | **Hearing-communication** | | | **Vision-social awareness** | | | **Personal-social** | | | **Emotional-behavioural-attention** | | | **Speech-language-communication** | | |
|  | Unadjusted | Confounder adjusted | Fully adjusted | Unadjusted | Confounder adjusted | Fully adjusted | Unadjusted | Confounder adjusted | Fully adjusted | Unadjusted | Confounder adjusted | Fully adjusted | Unadjusted | Confounder adjusted | Fully adjusted | Unadjusted | Confounder adjusted | Fully adjusted |
| **Hospital-diagnosed prenatal infection(s)** |  |  |  |  |  |  |  |  |  |  |  |  |  |  |  |  |  |  |
| *[No]* |  |  |  |  |  |  |  |  |  |  |  |  |  |  |  |  |  |  |
| *Yes* | 1.35*  (1.06-1.74) | 1.30*  (1.01-1.67) | 1.30*  (1.01-1.67) | 1.39  (0.86-2.25) | 1.35  (0.83-2.20) | 1.33  (0.82-2.17) | 1.50**  (1.11-2.01) | 1.49**  (1.11-2.01) | 1.46*  (1.08-1.96) | 1.50***  (1.29-1.74) | 1.39***  (1.20-1.62) | 1.34***  (1.15-1.56) | 1.54***  (1.38-1.71) | 1.42***  (1.27-1.58) | 1.36***  (1.22-1.52) | 1.43***  (1.29-1.58) | 1.36***  (1.23-1.50) | 1.33***  (1.20-1.47) |
| **Maternal age at time of birth** |  |  |  |  |  |  |  |  |  |  |  |  |  |  |  |  |  |  |
| *<20* |  | 1.20  (0.91-1.58) | 1.22  (0.93-1.60) |  | 1.65*  (1.02-2.68) | 1.63*  (1.01-2.65) |  | 1.00  (0.68-1.46) | 0.99  (0.68-1.45) |  | 1.17  (0.99-1.38) | 1.10  (0.93-1.31) |  | 1.75***  (1.58-1.95) | 1.65***  (1.48-1.84) |  | 1.20**  (1.07-1.34) | 1.16*  (1.03-1.29) |
| *[20-35]* |  |  |  |  |  |  |  |  |  |  |  |  |  |  |  |  |  |  |
| *>35* |  | 1.18  (0.99-1.41) | 1.78  (0.99-1.40) |  | 1.01  (0.71-1.42) | 1.01  (0.71-1.42) |  | 1.15  (0.94-1.42) | 1.15  (0.93-1.42) |  | 0.93  (0.83-1.05) | 0.93  (0.83-1.05) |  | 0.83***  (0.76-0.90) | 0.83***  (0.76-0.91) |  | 0.94  (0.88-1.01) | 0.95  (0.88-1.02) |
| **SIMD quintile** |  |  |  |  |  |  |  |  |  |  |  |  |  |  |  |  |  |  |
| *1 (most deprived)* |  | 1.66*** (1.35-2.05) | 1.68***  (1.36-2.07) |  | 1.16  (0.79-1.71) | 1.14  (0.77-1.69) |  | 1.03  (0.81-1.30) | 0.99  (0.78-1.26) |  | 1.71***  (1.51-1.93) | 1,59***  (1.40-1.80) |  | 1.78***  (1.63-1.94) | 1.63***  (1.50-1.79) |  | 1.31***  (1.22-1.41) | 1.26***  (1.17-1.36) |
| *2 (more deprived)* |  | 1.55*** (1.23-1.96) | 1.56***  (1.24-1.97) |  | 1.36  (0.88-2.08) | 1.35  (0.88-2.07) |  | 1.05  (0.81-1.30) | 1.03  (0.79-1.35) |  | 1.30***  (1.13-1.50) | 1.28**  (1.11-1.47) |  | 1.32***  (1.20-1.46) | 1.29***  (1.17-1.43) |  | 1.11*  (1.02-1.21) | 1.09*  (1.01-1.20) |
| *[3 (medium deprived)]* |  |  |  |  |  |  |  |  |  |  |  |  |  |  |  |  |  |  |
| *4 (less deprived)* |  | 1.09  (0.83-1.43) | 1.09  (0.83-1.43) |  | 0.94  (0.57-1.57 | 0.95  (0.57-1.58) |  | 0.93  (0.69-1.26) | 0.95  (0.71-1.28) |  | 0.78**  (0.65-0.93) | 0.80*  (0.68-0.97) |  | 0.82**  (0.73-0.93) | 0.86*  (0.76-0.97) |  | 0.80***  (0.73-0.89) | 0.82***  (0.74-0.91) |
| *5 (least deprived)* |  | 0.99  (0.76-1.31) | 0.99  (0.75-1.30) |  | 1.09  (0.67-1.78) | 1.11  (0.68-1.80) |  | 0.91  (0.68-1.23) | 0.93  (0.69-1.26) |  | 0.57***  (0.47-0.68) | 0.60***  (0.49-0.72) |  | 0.65***  (0.57-0.73) | 0.69***  (0.61-0.79) |  | 0.61***  (0.55-0.68) | 0.63***  (0.56-0.70) |
| **Sex of child** |  |  |  |  |  |  |  |  |  |  |  |  |  |  |  |  |  |  |
| *[Male]* |  |  |  |  |  |  |  |  |  |  |  |  |  |  |  |  |  |  |
| *Female* |  |  | 0.76***  (0.67-0.87) |  |  | 0.80  (0.63-1.03) |  |  | 0.82*  (0.70-0.96) |  |  | 0.41***  (0.38-0.44) |  |  | 0.49***  (0.46-0.52) |  |  | 0.38***  (0.36-0.40) |
| **Maternal history of mental health hospital admissions** |  |  |  |  |  |  |  |  |  |  |  |  |  |  |  |  |  |  |
| *[No]* |  |  |  |  |  |  |  |  |  |  |  |  |  |  |  |  |  |  |
| *Yes* |  |  | 1.46  (0.96-2.21) |  |  | 1.41  (0.61-3.23) |  |  | 2.05**  (1.32-3.18) |  |  | 1.55***  (1.22-1.98) |  |  | 1.50***  (1.25-1.80) |  |  | 1.31**  (1.10-1.56) |
| **Maternal prenatal smoking** |  |  |  |  |  |  |  |  |  |  |  |  |  |  |  |  |  |  |
| *[No]* |  |  |  |  |  |  |  |  |  |  |  |  |  |  |  |  |  |  |
| *Yes* |  |  | 0.89  (0.74-1.07) |  |  | 1.15  (0.81-1.62) |  |  | 1.20  (0.96-1.48) |  |  | 1.72***  (1.56-1.89) |  |  | 1.94***  (1.81-2.08) |  |  | 1.45***  (1.35-1.56) |

*Notes*: Reference categories are shown in square brackets. *p<0.05, **p<0.01, ***p<0.001.

**Table S6b.** Odds ratios (95% CIs) for unadjusted, confounder adjusted and fully adjusted associations between receipt of infection-related prescription(s) during pregnancy and having specific types of childhood developmental concerns identified by health visitors.

|  | **Type of childhood developmental concern identified** | | | | | | | | | | | | | | | | | |
| --- | --- | --- | --- | --- | --- | --- | --- | --- | --- | --- | --- | --- | --- | --- | --- | --- | --- | --- |
|  | **6-8 weeks child health review** | | | | | | | | | **27-30 months child health review** | | | | | | | | |
|  | **Gross motor** | | | **Hearing-communication** | | | **Vision-social awareness** | | | **Personal-social** | | | **Emotional-behavioural-attention** | | | **Speech-language-communication** | | |
|  | Unadjusted | Confounder adjusted | Fully adjusted | Unadjusted | Confounder adjusted | Fully adjusted | Unadjusted | Confounder adjusted | Fully adjusted | Unadjusted | Confounder adjusted | Fully adjusted | Unadjusted | Confounder adjusted | Fully adjusted | Unadjusted | Confounder adjusted | Fully adjusted |
| **Receipt of infection-related prescription(s) during pregnancy** |  |  |  |  |  |  |  |  |  |  |  |  |  |  |  |  |  |  |
| *[No]* |  |  |  |  |  |  |  |  |  |  |  |  |  |  |  |  |  |  |
| *Yes* | 1.08  (0.94-1.24) | 1.05  (0.91-1.21) | 1.05  (0.91-1.21 | 1.13  (0.86-1.48) | 1.08  (0.82-1.43) | 1.08  (0.82-1.43) | 0.92  (0.78-1.11) | 0.93  (0.77-1.11) | 0.92  (0.77-1.10) | 1.19***  (1.09-1.29) | 1.12**  (1.03-1.22) | 1.12*  (1.03-1.22) | 1.26***  (1.19-1.33) | 1.15***  (1.08-1.22) | 1.15***  (1.08-1.23) | 1.02  (0.97-1.08) | 0.97  (0.92-1.02) | 0.97  (0.92-1.03) |
| **Maternal age at time of birth** |  |  |  |  |  |  |  |  |  |  |  |  |  |  |  |  |  |  |
| *<20* |  | 1.20  (0.90-1.58) | 1.22  (0.92-1.60) |  | 1.64  (1.00-2.69) | 1.62  (0.99-2.65) |  | 1.04  (0.71-1.52) | 1.03  (0.70-1.51) |  | 1.15  (0.97-1.36) | 1.08  (0.91-1.28) |  | 1.72***  (1.54-1.91) | 1.61***  (1.44-1.80) |  | 1.22***  (1.09-1.36) | 1.18**  (1.05-1.32) |
| *[20-35]* |  |  |  |  |  |  |  |  |  |  |  |  |  |  |  |  |  |  |
| *>35* |  | 1.18  (0.99-1.41) | 1.18  (0.99-1.40) |  | 1.01  (0.71-1.43) | 1.01  (0.71-1.43) |  | 1.15  (0.93-1.42) | 1.14  (093-1.41) |  | 0.93  (0.83-1.05) | 0.94  (0.83-1.05) |  | 0.83***  (0.76-0.91) | 0.84***  (0.77-0.91) |  | 0.94  (0.88-1.01) | 0.95  (0.88-1.02) |
| **SIMD quintile** |  |  |  |  |  |  |  |  |  |  |  |  |  |  |  |  |  |  |
| *1 (most deprived)* |  | 1.67***  (1.35-2.05) | 1.68***  (1.36-2.08) |  | 1.17  (0.79-1.72) | 1.14  (0.78-1.69) |  | 1.03  (0.82-1.30 | 1.00  (0.79-1.36) |  | 1.71***  (1.51-1.93) | 1.59***  (1.40-1.80) |  | 1.78***  (1.63-1.94) | 1.64***  (1.50-1.79) |  | 1.32***  (1.22-1.42) | 1.26***  (1.17-1.36) |
| *2 (more deprived)* |  | 1.56***  (1.23-1.96) | 1.56***  (1.24-1.97) |  | 1.36  (0.89-2.08) | 1.35  (0.88-2/07) |  | 1.05  (0.80-1.37) | 1.04  (0.80-1.36) |  | 1.30***  (1.13-1.50) | 1.28**  (1.11-1.47) |  | 1.32***  (1.20-1.46) | 1.29***  (1.17-1.43) |  | 1.11*  (1.02-1.21) | 1.10*  (1.01-1.20) |
| *[3 (medium deprived)]* |  |  |  |  |  |  |  |  |  |  |  |  |  |  |  |  |  |  |
| *4 (less deprived)* |  | 1.09  (0.83-1.43) | 1.09  (0.83-1.43) |  | 0.95  (0.57-1.57) | 0.95  (0.57-1.59) |  | 0.93  (0.69-1.26) | 0.94  (0.70-1.27) |  | 0.78**  (0.66-0.93) | 0.81*  (0.68-0.96) |  | 0.83**  (0.73-0.93) | 0.86*  (0.76-0.97) |  | 0.80***  (0.73-0.89) | 0.82***  (0.74-0.91) |
| *5 (least deprived)* |  | 0.99  (0.75-1.30) | 0.99  (0.75-1.30) |  | 1.09  (0.66-1.77) | 1.11  (0.68-1.80) |  | 0.90  (0.67-1.22) | 0.92  (0.69-1.25) |  | 0.56***  (0.47-0.68) | 0.60***  (0.49-0.72) |  | 0.65***  (0.57-0.74) | 0.69***  (0.61-0.79) |  | 0.61***  (0.55-0.68) | 0.62***  (0.56-0.69) |
| **Sex of child** |  |  |  |  |  |  |  |  |  |  |  |  |  |  |  |  |  |  |
| *[Male]* |  |  |  |  |  |  |  |  |  |  |  |  |  |  |  |  |  |  |
| *Female* |  |  | 0.76***  (0.67-0.87) |  |  | 0.80  (0.62-3.26) |  |  | 0.82*  (0.70-0.96) |  |  | 0.41***  (0.38-0.45) |  |  | 0.49***  (0.46-0.52) |  |  | 0.38***  (0.36-0.40) |
| **Maternal history of mental health hospital admissions** |  |  |  |  |  |  |  |  |  |  |  |  |  |  |  |  |  |  |
| *[No]* |  |  |  |  |  |  |  |  |  |  |  |  |  |  |  |  |  |  |
| *Yes* |  |  | 1.47  (0.97-2.23) |  |  | 1.42  (0.62-3.26) |  |  | 2.09**  1.35-3.21) |  |  | 1.57***  (1.23-1.99) |  |  | 1.51***  (1.26-1.81) |  |  | 1.32**  (1.11-1.58) |
| **Maternal prenatal smoking** |  |  |  |  |  |  |  |  |  |  |  |  |  |  |  |  |  |  |
| *[No]* |  |  |  |  |  |  |  |  |  |  |  |  |  |  |  |  |  |  |
| *Yes* |  |  | 0.90  (0.75-1.07) |  |  | 1.15  (0.82-1.63) |  |  | 1.21  (0.97-1.50) |  |  | 1.73***  (1.57-1.90) |  |  | 1.95***  (1.82-2.09) |  |  | 1.45***  (1.36-1.55) |

*Notes*: Reference categories are shown in square brackets. *p<0.05, **p<0.01, ***p<0.001.

**Table S7a. Odds ratios (95% CIs) for unadjusted, confounder adjusted and fully adjusted associations between hospital-diagnosed prenatal infections, by trimester, and having any (i.e. at least one) childhood developmental concerns identified by health visitors.**

|  | **Having any (i.e. at least one) childhood developmental concerns identified** | | | | | | | | |
| --- | --- | --- | --- | --- | --- | --- | --- | --- | --- |
|  | **Timing of hospital-diagnosed prenatal infection** | | | | | | | | |
|  | **Trimester 1** | | | **Trimester 2** | | | **Trimester 3** | | |
|  | Unadjusted | Confounder adjusted | Fully adjusted | Unadjusted | Confounder adjusted | Fully adjusted | Unadjusted | Confounder adjusted | Fully adjusted |
| **Hospital-diagnosed prenatal infection(s)** |  |  |  |  |  |  |  |  |  |
| *[No]* |  |  |  |  |  |  |  |  |  |
| *Yes* | 1.24  (0.95-1.60) | 1.13  (0.87-1.46) | 1.11  (0.84-1.45) | 1.53***  (1.23-1.89) | 1.39**  (1.12-1.72) | 1.34*  (1.07-1.67) | 1.45***  (1.32-1.59) | 1.37***  (1.24-1.50) | 1.33***  (1.21-1.47) |
| **Maternal age at time of birth** |  |  |  |  |  |  |  |  |  |
| *<20* |  | 1.56***  (1.43-1.71) |  |  | 1.56***  (1.43-1.70) | 1.50***  (1.37-1.64) |  | 1.55***  (1.42-1.69) | 1.49***  (1.36-1.64) |
| *[20-35]* |  |  |  |  |  |  |  |  |  |
| *>35* |  | 0.94*  (0.88-0.99) |  |  | 0.94*  (0.88-0.99) | 0.94  (0.89-1.00) |  | 0.94*  (0.88-0.99) | 0.94  (0.89-1.00) |
| **SIMD quintile** |  |  |  |  |  |  |  |  |  |
| *1 (most deprived)* |  | 1.47***  (1.38-1.56) | 1.39***  (1.31-1.48) |  | 1.47***  (1.38-1.56) | 1.39***  (1.31-1.48) |  | 1.47***  (1.38-1.56) | 1.39***  (1.31-1.48) |
| *2 (more deprived)* |  | 1.20***  (1.12-1.29) | 1.18***  (1.31-1.48) |  | 1.20***  (1.12-1.29) | 1.18***  (1.10-1.27) |  | 1.20***  (1.12-1.29) | 1.18***  (1.10-1.27) |
| *[3 (medium deprived)]* |  |  |  |  |  |  |  |  |  |
| *4 (less deprived)* |  | 0.81***  (0.75-0.89) | 0.84***  (0.76-0.91) |  | 0.82***  (0.75-0.89) | 0.83***  (0.77-0.90) |  | 0.82***  (0.75-0.89) | 0.83***  (0.76-0.91) |
| *5 (least deprived)* |  | 0.64***  (0.59-0.70) | 0.67***  (0.61-0.73) |  | 0.64***  (0.59-0.70) | 0.67***  (0.61-0.73) |  | 0.65***  (0.59-0.70) | 0.67***  (0.61-0.73) |
| **Sex of child** |  |  |  |  |  |  |  |  |  |
| *[Male]* |  |  |  |  |  |  |  |  |  |
| *Female* |  |  | 0.46***  (0.44-0.48) |  |  | 0.50***  (0.44-0.48) |  |  | 0.46***  (0.44-0.48) |
| **Maternal history of mental health hospital admissions** |  |  |  |  |  |  |  |  |  |
| *[No]* |  |  |  |  |  |  |  |  |  |
| *Yes* |  |  | 1.50***  (1.29-1.73) |  |  | 1.50***  (1.29-1.73) |  |  | 1.48***  (1.28-1.73) |
| **Maternal prenatal smoking** |  |  |  |  |  |  |  |  |  |
| *[No]* |  |  |  |  |  |  |  |  |  |
| *Yes* |  |  | 1.64***  (1.56-1.74) |  |  | 1.64***  (1.55-1.74) |  |  | 1.64***  (1.55-1.73) |

*Notes*: Reference categories are shown in square brackets. Childhood developmental concerns include those measured at both 6-8 weeks and 27-30 months routine child health visits. *p<0.05, **p<0.01, ***p<0.001.

**Table S7b.** Odds ratios (95% CIs) for unadjusted, confounder adjusted and fully adjusted associations between receipt of infection-related prescription(s), by trimester, and having any (i.e. at least one) childhood developmental concerns identified by health visitors.

|  | **Having any (i.e. at least one) childhood developmental concerns identified** | | | | | | | | |
| --- | --- | --- | --- | --- | --- | --- | --- | --- | --- |
|  | **Timing of hospital-diagnosed prenatal infection** | | | | | | | | |
|  | **Trimester 1** | | | **Trimester 2** | | | **Trimester 3** | | |
|  | Unadjusted | Confounder adjusted | Fully adjusted | Unadjusted | Confounder adjusted | Fully adjusted | Unadjusted | Confounder adjusted | Fully adjusted |
| **Receipt of infection-related prescription(s)** |  |  |  |  |  |  |  |  |  |
| *[No]* |  |  |  |  |  |  |  |  |  |
| *Yes* | 1.20***  (1.12-1.27) | 1.10**  (1.03-1.18) | 1.09*  (1.02-1.16) | 1.15***  (1.08-1.23) | 1.06  (0.99-1.14) | 1.06*  (1.00-1.14) | 1.11***  (1.05-1.17) | 1.05  (0.99-1.11) | 1.04  (0.99-1.10) |
| **Maternal age at time of birth** |  |  |  |  |  |  |  |  |  |
| *<20* |  | 1.54***  (1.41-1.69) | 1.49***  (1.35-1.63) |  | 1.55***  (1.42-1.70) | 1.49***  (1.36-1.63) |  | 1.55***  (1.42-1.70) | 1.49***  (1.36-1.64) |
| *[20-35]* |  |  |  |  |  |  |  |  |  |
| *>35* |  | 0.94*  (0.89-0.99) | 0.94  (0.89-1.00) |  | 0.94*  (0.89-0.99) | 0.94  (0.89-1.00) |  | 0.94*  (0.89-0.99) | 0.94*  (0.89-0.99) |
| **SIMD quintile** |  |  |  |  |  |  |  |  |  |
| *1 (most deprived)* |  | 1.47***  (1.38-1.56) | 1.39***  (1.30-1.48) |  | 1.47***  (1.38-1.56) | 1.39***  (1.31-1.48) |  | 1.47***  (1.38-1.56) | 1.39***  (1.31-1.48) |
| *2 (more deprived)* |  | 1.20***  (1.12-1.29) | 1.18***  (1.10-1.27) |  | 1.20***  (1.12-1.29) | 1.18***  (1.10-1.27) |  | 1.20***  (1.12-1.29) | 1.18***  (1.10-1.27) |
| *[3 (medium deprived)]* |  |  |  |  |  |  |  |  |  |
| *4 (less deprived)* |  | 0.82***  (0.75-0.88) | 0.84***  (0.77-0.90) |  | 0.82***  (0.75-0.88) | 0.84***  (0.77-0.90) |  | 0.82***  (0.75-0.89) | 0.84***  (0.77-0.91) |
| *5 (least deprived)* |  | 0.64***  (0.59-0.70) | 0.67***  (0.61-0.73) |  | 0.64***  (0.59-0.70) | 0.67***  (0.61-0.72) |  | 0.64***  (0.59-0.70) | 0.67***  (0.61-0.73) |
| **Sex of child** |  |  |  |  |  |  |  |  |  |
| *[Male]* |  |  |  |  |  |  |  |  |  |
| *Female* |  |  | 0.46***  (0.44-0.48) |  |  | 0.46***  (0.44-0.48) |  |  | 0.46***  (0.44-0.48) |
| **Maternal history of mental health hospital admissions** |  |  |  |  |  |  |  |  |  |
| *[No]* |  |  |  |  |  |  |  |  |  |
| *Yes* |  |  | 1.49***  (1.28-1.73) |  |  | 1.49***  (1.29-1.73) |  |  | 1.50***  (1.29-1.73) |
| **Maternal prenatal smoking** |  |  |  |  |  |  |  |  |  |
| *[No]* |  |  |  |  |  |  |  |  |  |
| *Yes* |  |  | 1.64***  (1.55-1.74) |  |  | 1.64***  (1.56-1.74) |  |  | 1.64***  (1.55-1.74) |

*Notes*: Reference categories are shown in square brackets. *p<0.05, **p<0.01, ***p<0.001

**Table S8a.** Odds ratios (95% CIs) for fully adjusted associations between prenatal infections (with month of childbirth excluded) and having any (i.e. at least one) adverse childhood development outcome.

|  | **Having any (i.e. at least one) adverse childhood development outcome** |
| --- | --- |
| **Hospital-diagnosed prenatal infection** |  |
| *[No]* |  |
| *Yes* | 1.38*** (1.23-1.56) |
| **Receipt of infection-related prescription(s) during pregnancy** |  |
| *[No]* |  |
| *Yes* | 1.05 (1.00-1.10) |

*Notes*: models fully adjust for maternal age at time of birth, SIMD quintile, sex of child, maternal history of mental health hospital admissions and maternal prenatal smoking. Reference categories are shown in square brackets. *p<0.05, **p<0.01, ***p<0.001.

**Table S8b. Odds ratios (95% CIs) for fully adjusted associations between prenatal infections (with month of childbirth excluded) and having a specific type of adverse childhood development outcome.**

|  | **Type of adverse childhood development outcome** | | | | | |
| --- | --- | --- | --- | --- | --- | --- |
|  | **6-8 weeks child health review** | | | **27-30 months child health review** | | |
|  | **Gross motor** | **Hearing-communication** | **Vision-social awareness** | **Personal-social** | **Emotional-behavioural-attention** | **Speech-language-communication** |
| **Hospital-diagnosed prenatal infection** |  |  |  |  |  |  |
| *[No]* |  |  |  |  |  |  |
| *Yes* | 1.21 (0.86-1.72) | 1.38 (0.73-2.64) | 1.67** (1.14-2.43) | 1.29* (1.04-1.58) | 1.42*** (1.23-1.64) | 1.45*** (1.27-1.67) |
| **Receipt of infection-related prescription(s) during pregnancy** |  |  |  |  |  |  |
| *[No]* |  |  |  |  |  |  |
| *Yes* | 1.07 (0.93-1.24) | 1.08 (0.80-1.44) | 1.00 (0.83-1.20) | 1.16* (1.06-1.27) | 1.16*** (1.08-1.23) | 0.99 (0.93-1.05) |

*Notes*: models fully adjust for maternal age at time of birth, SIMD quintile, sex of child, maternal history of mental health hospital admissions and maternal prenatal smoking. Reference categories are shown in square brackets. *p<0.05, **p<0.01, ***p<0.00
